# Favorable Long-Term Outcomes of Endoscopic Submucosal Dissection for Differentiated-Type-Predominant Early Gastric Cancer with Histological Heterogeneity

**DOI:** 10.3390/jcm9041064

**Published:** 2020-04-09

**Authors:** Tae-Se Kim, Hyeong Chan Shin, Byung-Hoon Min, Kyoung-Mee Kim, Yang Won Min, Hyuk Lee, Jun Haeng Lee, Poong-Lyul Rhee, Jae J. Kim

**Affiliations:** 1 Department of Medicine, Samsung Medical Center, Sungkyunkwan University School of Medicine, Seoul 06351, Korea; tsk1029@naver.com (T.-S.K.); yangwon.min@samsung.com (Y.W.M.); lhyuk.lee@samsung.com (H.L.); jh2145.lee@samsung.com (J.H.L.); pl.rhee@samsung.com (P.-L.R.); jaej.kim@samsung.com (J.J.K.); 2 Department of Pathology and Translational Genomics, Samsung Medical Center, Sungkyunkwan, University School of Medicine, Seoul 06351, Korea; ckslcksl@naver.com (H.C.S.); kkmkys@skku.edu (K.-M.K.)

**Keywords:** early gastric cancer, histological heterogeneity, endoscopic submucosal dissection

## Abstract

It remains unclear whether endoscopic submucosal dissection (ESD) can be indicated for differentiated-type-predominant early gastric cancer mixed with a minor undifferentiated component (EGC with histological heterogeneity (HH)). Here, we reviewed and compared clinicopathologic characteristics and long-term outcomes of ESD of 257 patients with EGC-HH and those of 2386 patients with pure differentiated-type EGC (PuD-EGC)**.** After ESD, EGC-HH was managed in the same way as PuD-EGC. EGC-HHs were significantly associated with larger tumor size, more frequent submucosal invasion, and lymphovascular invasion compared to PuD-EGCs. Despite these aggressive features of EGC-HH, no local recurrence or gastric cancer-related death occurred during a median of 58 months of follow up after ESD for EGC-HH, if curative resection was achieved. After curative ESD for EGC-HH, six patients had metachronous recurrence (5.0%) and one patient underwent extragastric recurrence in a regional lymph node (0.8%). All these recurrence cases were curatively treated with ESD or gastrectomy. For patients with EGC-HH, five-year overall survival and recurrence-free survival rates after curative ESD were 97.0% and 94.8%, respectively, which were comparable to those of patients with PuD-EGC. In conclusion, ESD showed favorable long-term outcomes after curative resection and may be an acceptable treatment option for EGC-HH meeting curative endoscopic resection criteria.

## 1. Introduction

In current Korean and Japanese guidelines, differentiated-type-predominant early gastric cancer (EGC) mixed with a minor undifferentiated component is regarded as differentiated-type EGC [1,2]. This differentiated-type-predominant EGC with histological heterogeneity (EGC-HH) has more aggressive clinicopathologic behavior and a higher risk of lymph node (LN) metastasis compared to pure differentiated-type EGC (PuD-EGC) without an undifferentiated component [3,4,5,6,7]. However, previous studies based on surgical specimens have consistently reported the minimal risk of LN metastasis in EGC-HH, provided that the EGC-HH met the curative endoscopic resection criteria for tumors of absolute or expanded indications [3,5,6,7]. Our previous study on the outcomes of endoscopic submucosal dissection (ESD) for EGC-HH also supported the minimal risk of LN metastasis in EGC-HH meeting the curative endoscopic resection criteria [4]. In that study, no LN metastasis or gastric cancer-related death was observed after ESD for EGC-HH meeting the curative endoscopic resection criteria. However, our previous study was limited due to its relatively short follow-up duration for recurrence (median 27.5 months, range 2–63 months). To guarantee the favorable long-term outcomes of ESD for EGC-HH, studies with an approximately five-year follow up are required. 

According to current Japanese gastric cancer treatment guidelines, ESD for EGC-HH is still regarded as non-curative if areas of an undifferentiated-type carcinoma exceed 20 mm in the intramucosal tumor without an ulcer, or if the undifferentiated-type component invades the submucosal layer in a submucosal (SM)1 tumor ≤3 cm [1]. To date, few studies have evaluated the outcomes of ESD for EGC-HH with the above-mentioned features, and supporting evidence for these criteria is still lacking. 

In the present study, we aimed to elucidate the long-term outcomes of ESD for EGC-HH compared to those of PuD-EGC. We also performed a detailed pathologic review of ESD specimens of EGC-HH in terms of the extent and invasion depth of the undifferentiated-type component.

## 2. Materials and Methods

### 2.1. Patients 

Between April 2007 and April 2014, 2782 patients with 2909 differentiated-type EGCs (well- or moderately-differentiated EGC or papillary EGC) underwent their first ESD at Samsung Medical Center [1,8]. Differentiated-type EGCs included PuD-EGC without an undifferentiated component and EGC-HH mixed with a minor undifferentiated component (poorly differentiated EGC or signet ring cell EGC) accounting for less than 50% of the tumor area [1]. Patients who were diagnosed to have undifferentiated-type EGC (poorly differentiated EGC or signet ring cell EGC accounting for more than 50% of the tumor area) based on ESD pathology were excluded from the study population [1]. Among patients undergoing ESD for differentiated-type EGCs, the following patients were excluded: (1) 21 patients with 22 EGCs arising in the remnant stomach; (2) 2 patients with 2 EGCs arising in the gastric tube after esophagectomy; and (3) 116 patients with multiple EGCs including 12 patients having concurrent EGC-HH and PuD-EGC. After exclusion, a total of 2386 patients with 2386 PuD-EGCs and 257 patients with 257 EGC-HHs were finally enrolled in the study.

In our institution, the routine pre-ESD diagnostic work-up of EGC patients included esophagogastroduodenoscopy (EGD) with biopsy and abdominal computed tomography (CT). ESD was performed when LN metastasis was not detected on abdominal CT. 

Clinicopathologic data and outcomes after ESD were obtained through a retrospective review of medical records from the intranet resources of Samsung Medical Center. To ensure five-year follow up, the censoring date was set as April 30, 2019. All enrolled patients provided written informed consent according to our institutional guidelines. The study protocol was approved by the institutional review board of Samsung Medical Center (approval number 2018-08-143).

### 2.2. Definitions

En bloc resection was defined as resection of the tumor in one piece without endoscopic evidence of a residual tumor. R0 resection was defined as resection of the tumor with no cancer cell involvement of the lateral and vertical margins. The resection was defined as curative when all the following criteria were fulfilled [1]: PuD-EGC without an undifferentiated component and EGC-HH mixed with a minor undifferentiated component, en bloc resection, negative lateral resection margins, negative vertical resection margins, and no lymphovascular invasion: (1) tumor size ≤2 cm, mucosal cancer, no ulcer in the tumor; (2) tumor size >2 cm, mucosal cancer, no ulcer in the tumor; (3) tumor size ≤3 cm, mucosal cancer, ulcer in the tumor; or (4) tumor size ≤3 cm, SM1 cancer (submucosal invasion depth <500 µm from the muscularis mucosa layer). Non-curative ESD was defined when any of the above curative resection criteria was not met. 

Local recurrence was defined when the cancer was detected at the primary ESD site in a follow-up EGD after R0 resection. Metachronous recurrence was defined when the cancer was detected at a location other than the primary ESD site at least 12 months after ESD. When such a lesion was detected within 12 months after ESD, it was defined as a synchronous lesion. 

### 2.3. Histopathological Evaluation 

Histopathological evaluation of the ESD specimens in our institution has been described in detail elsewhere [4,9,10]. For EGC-HH cases meeting the curative endoscopic resection criteria for tumors of expanded indications [1], we performed a detailed pathologic review of the undifferentiated-type component, including its proportion in the tumor, its total length, and presence or absence in the submucosal invasion area.

### 2.4. Follow Up after Endoscopic Submucosal Dissection

EGD along with a biopsy was performed two months after ESD to confirm the healing of the artificial ulcer and to exclude the possibility of any recurrence. Thereafter, EGD with a biopsy and abdominal CT were carried out at six-month intervals for three years, and then annually from the fourth to the fifth year after ESD.

### 2.5. Statistical Analysis

Categorical variables were analyzed using the chi-square test or Fisher’s exact test. Continuous variables were analyzed using the Student’s t-test or the Mann–Whitney test. Data on overall survival were obtained using the national registry of medical insurance. Overall survival was measured from the date of ESD to the date of death from any cause or to the censoring date (April 30, 2019). Recurrence-free survival was determined from the date of ESD to the first local, metachronous, or extragastric recurrence or death with evidence of recurrence. Survival curves were plotted with the Kaplan–Meier method, and the difference between the curves was tested using the log-rank test. The following patients were excluded from calculating the survival rate: (1) patients undergoing gastrectomy after ESD; (2) patients not undergoing follow-up examination after ESD; (3) patients with a synchronous lesion detected within 12 months after ESD.

A *p*-value less than 0.05 was considered statistically significant. All analyses were performed with SPSS software version 25.0 (IBM SPSS Statistics for Windows, Version 25.0. Armonk, NY, USA: IBM Corp.).

## 3. Results

### 3.1. Comparison of Clinicopathologic Characteristics of Differentiated-Type-Predominant Early Gastric Cancers with Histological Heterogeneity versus Pure Differentiated-Type Early Gastric Cancer

EGC-HH accounted for 9.7% of all differentiated-type EGCs enrolled in this study. Among 257 EGC-HH cases, 239 (93.0%) were mixed with a poorly differentiated carcinoma component, and only 18 cases (7.0%) showed a signet ring cell carcinoma component. The proportion of undifferentiated component was as follows: 1–9% in 86 cases, 10–19% in 86 cases, 20–29% in 38 cases, 30–39% in 28 cases, and 40–49% in 19 cases.

Table 1 summarizes and compares the clinicopathologic characteristics of patients with EGC-HH versus PuD-EGC. EGC-HHs were significantly associated with larger tumor size, more frequent submucosal invasion, moderately-differentiated histology, lymphovascular invasion, and tumor involvement of the resection margin compared to PuD-EGC.

### 3.2. Comparison of Short-Term Outcomes of Endoscopic Submucosal Dissection of Differentiated-Type-Predominant Early Gastric Cancers with Histological Heterogeneity versus Pure Differentiated-Type Early Gastric Cancer 

Table 2 summarizes the short-term outcomes of ESD for EGC-HH versus PuD-EGC. The R0 resection and en bloc with R0 resection rates for EGC-HH were 85.6% and 84.0%, respectively, which were significantly lower than those for PuD-EGC (95.8% and 94.7%, respectively). The curative resection rates were 49.0% and 86.2% in EGC-HH and PuD-EGC, respectively. The major reasons for non-curative resection in ESD for EGC-HH were lymphatic invasion and deep submucosal invasion (SM2 or SM3), which were present in 57.3% (75/131) and 55.0 % (72/131) of non-curative resection cases, respectively.

Perforation and bleeding rates in EGC-HH were 3.1% and 5.1%, respectively, and those in PuD-EGC were 1.9% and 4.4%, respectively.

### 3.3. Recurrence Pattern after Endoscopic Submucosal Dissection for Differentiated-Type-Predominant Early Gastric Cancers with Histological Heterogeneity 

**Figure 1:**
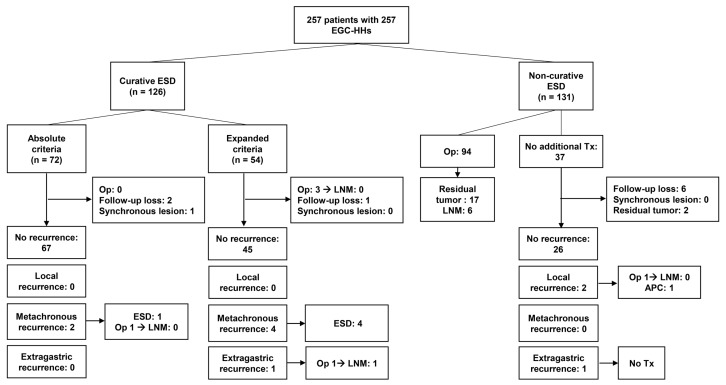
Flowchart for the outcomes of endoscopic submucosal dissection for differentiated-type-predominant early gastric cancer with histological heterogeneity. EGC-HH, differentiated-type-predominant early gastric cancer with histological heterogeneity, ESD, endoscopic submucosal dissection; Op, operation; LNM, lymph node metastasis; Tx, treatment; APC, argon plasma coagulation.

Figure 1 displays the follow-up outcomes of enrolled patients with EGC-HH. Patients were followed up with EGD and abdominal CT for local, metachronous, and extragastric recurrence. 

After curative ESD for EGC-HH, there were six metachronous recurrences (5.0%, 6/119) and one extragastric recurrence (0.8%, 1/119) (Figure 1). Five patients with metachronous recurrence were curatively treated with ESD. One patient with metachronous recurrence underwent gastrectomy because the recurred tumor was found in an advanced stage. After surgery, no LN metastasis was found in the surgical specimen of this patient. No extragastric recurrence occurred after curative ESD in patients with EGC-HH meeting absolute endoscopic resection criteria. However, one patient with EGC-HH meeting expanded criteria underwent extragastric recurrence in a regional LN 49 months after curative ESD. The tumor treated with curative ESD was confined to the muscularis mucosa layer, and the size of the tumor was 40 mm. The length of the undifferentiated component was 8 mm. For extragastric recurrence, this patient underwent curative radical gastrectomy, and a pathology review revealed LN metastasis in one regional LN. This patient was alive on the censoring date without gastric cancer recurrence. During the follow-up period, no gastric cancer-related death occurred in patients with EGC-HH after curative ESD. 

**Figure 2:**
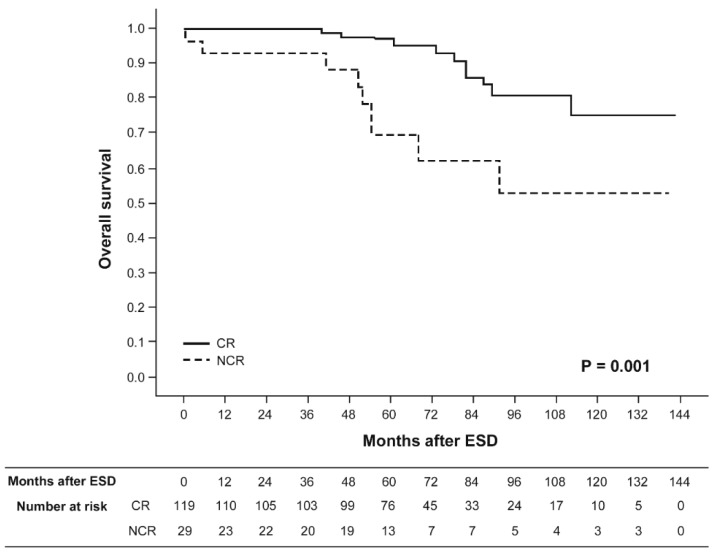
Kaplan–Meier overall survival curves for patients with differentiated-type-predominant early gastric cancer with histological heterogeneity undergoing endoscopic submucosal dissection. Solid line, patients treated with curative endoscopic resection (CR). Dotted line, patients undergoing non-curative endoscopic resection (NCR).

Among 131 patients undergoing non-curative ESD, however, three gastric cancer related deaths occurred 52.4, 56.7, and 76.4 months after non-curative ESD. After non-curative ESD, gastrectomy was performed in 95 patients, and LN metastasis was found in six (6.3%). The five-year overall survival rate of patients undergoing non-curative ESD was 69.6%, which was significantly lower than the survival rate of 97.0% in patients treated with curative ESD (Figure 2).

### 3.4. Comparison of Long-Term Outcomes of Endoscopic Submucosal Dissection of Differentiated-Type-Predominant Early Gastric Cancers with Histological Heterogeneity versus Pure Differentiated-Type Early Gastric Cancer

**Figure 3:**
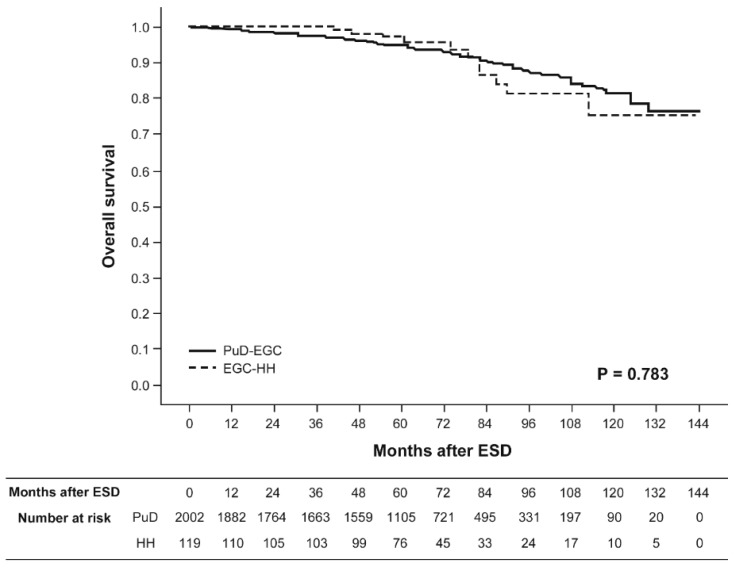
Kaplan–Meier overall survival curves for patients undergoing curative endoscopic submucosal dissection. Solid line, pure differentiated-type early gastric cancer (PuD-EGC); dotted line, differentiated-type-predominant early gastric cancer with histological heterogeneity (EGC-HH).

**Figure 4:**
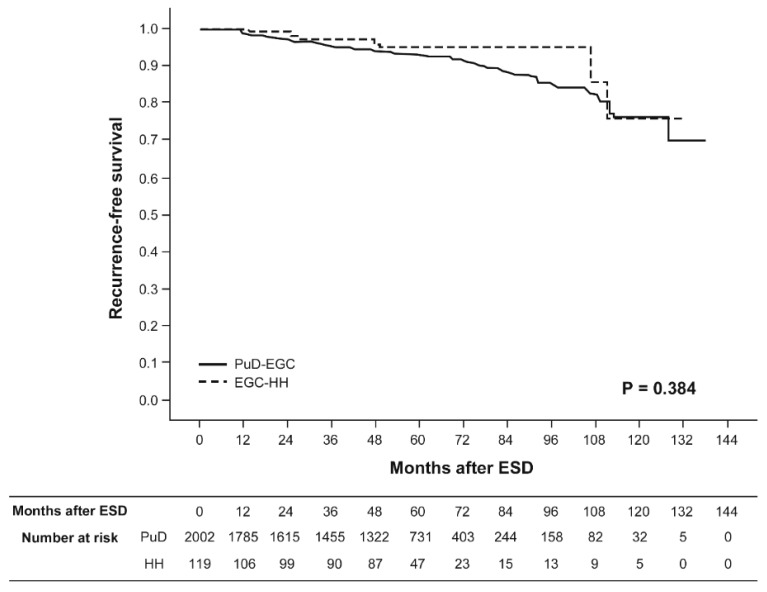
Kaplan–Meier recurrence-free survival curves for patients undergoing curative endoscopic submucosal dissection. Solid line, pure differentiated-type early gastric cancer (PuD-EGC); dotted line, differentiated-type-predominant early gastric cancer with histological heterogeneity (EGC-HH).

Figure 3 and Figure 4 show the Kaplan–Meier overall survival and recurrence-free survival curves for patients with EGC-HH versus PuD-EGC who were treated with curative ESD, respectively. The median follow-up duration for recurrence after curative ESD was 58 months (range 1–131 months) for patients with EGC-HH and 58 months (range 1–137 months) for patients with PuD-EGC, respectively. The five-year overall survival rates after curative ESD were 97.0% and 94.8% for patients with EGC-HH and PuD-EGC, respectively. The five-year recurrence-free survival rates after curative ESD were 94.8% and 93.2% for patients with EGC-HH and PuD-EGC, respectively. Neither overall survival nor recurrence-free survival rates showed a statistically significant difference between the two groups. Extragastric recurrence rates after curative ESD were 0.8% and 0.1% for patients with EGC-HH and PuD-EGC, respectively.

### 3.5. Pathology Review of Undifferentiated-Type Component in Differentiated-Type-Predominant Early Gastric Cancer with Histological Heterogeneity

For 54 EGC-HHs meeting the curative endoscopic resection criteria of expanded indications, we performed a detailed pathologic review of the undifferentiated-type component. Pathologic slides were available for review in 51 cases.

Among 51 EGC-HHs, 41 were mucosal cancers and 10 were SM1 cancers. The median length of the undifferentiated component was 6 mm (range 1–24 mm). Three cases had areas of undifferentiated component that exceeded 20 mm in length. All three EGC-HH cases were confined to the mucosa layer. None of the three cases showed recurrence during the 63, 63, and 84 months of follow up after ESD, respectively (Table 3). Among 10 SM1 cancers, an undifferentiated component was present in the submucosal invasion area in only one case. The length of the undifferentiated component in the submucosal invasion area was 1 mm. This case was treated with an additional gastrectomy, and no LN metastasis was found in the surgical specimen (Table 3).

## 4. Discussion

To date, few studies have evaluated the long-term outcomes of ESD for EGC-HH. Therefore, it remains controversial whether ESD can be indicated for EGC-HH in the same way as PuD-EGC. In the present study, the long-term outcomes of ESD for EGC-HH were favorable despite its aggressive clinicopathologic features and low curative resection rate of less than 50%, which was mainly due to high lymphatic invasion and deep submucosal invasion rates. After curative ESD for EGC-HH, only one extragastric recurrence occurred in a regional LN during a median follow-up period of 58 months. No local recurrence or gastric cancer-related death occurred during the follow-up period after curative ESD for EGC-HH. In addition, both five-year overall survival and recurrence-free survival rates of patients with EGC-HH were comparable to those of patients with PuD-EGC once curative resection was achieved. 

In our previous study, no LN metastasis or extragastric recurrence was observed after ESD for EGC-HH meeting the curative endoscopic resection criteria [4]. However, with a longer follow up and a larger study population, we experienced one case of regional LN recurrence 49 months after curative ESD for intramucosal EGC-HH. Hanaoka et al. [11] also reported a case of regional LN recurrence 14 months after curative ESD for intramucosal EGC-HH, meeting the expanded curative resection criteria. Given that the incidence of extragastric recurrence was lower than 1% and the recurrences occurred in resectable regional LNs in both cases, careful follow up without additional gastrectomy may be an acceptable option after ESD for EGC-HH if curative resection is achieved. Periodic follow up with abdominal CT is required after ESD, especially for patients with EGC-HH meeting the expanded curative resection criteria. To the best of our knowledge, this is the first large-scale long-term follow-up study of ESD for EGC-HH. The present study has provided evidence for the promising role of endoscopic treatment for EGC-HH meeting the curative endoscopic resection criteria.

According to the current Japanese gastric cancer treatment guidelines, ESD for EGC-HH is regarded as non-curative if an undifferentiated-type component invades the submucosal layer in SM1 tumor ≤3 cm [1]. Jung et al. [12] and Miyahara et al. [13] reported that the presence of an undifferentiated component in the submucosal invasion area was a significant risk factor for LN metastasis based on surgical specimens. In the study by Jung et al. [12], LN metastasis was found in 36.4% (4/11) of SM1 EGC-HH with a minor poorly differentiated carcinoma component accounting for more than 5% of the tumor area. This study did not evaluate the LN metastasis rate in EGC-HH with SM1 invasion meeting the expanded curative resection criteria. Miyahara et al. [13] reported the LN metastasis rate in EGC-HH cases with deep submucosal invasion (submucosal invasion depth ≥500 μm from the muscularis mucosa). EGC-HH cases with SM1 invasion were not included in the study population. Therefore, it remains controversial whether the presence of an undifferentiated component in the submucosal invasion area leads to increased risk of LN metastasis in EGC-HH with SM1 invasion meeting the expanded curative resection criteria. ESD for EGC-HH is also regarded as non-curative if areas of undifferentiated-type carcinoma exceed 20 mm in the intramucosal tumor without an ulcer [1]. However, there has been little evidence to support this criteria. In the present study, three intramucosal EGC-HH cases showed areas of undifferentiated component that exceeded 20 mm in length. None of these cases showed recurrence during over five years of follow up after curative ESD. Further large studies are required to validate the current Japanese criteria of non-curative endoscopic resection for EGC-HH.

This study had several limitations. First, it was performed at a single tertiary referral center and had a retrospective design. Second, there was a potential for selection bias. Some patients with EGC-HH may have undergone surgical resection instead of ESD if the tumor included a high proportion of undifferentiated component and was diagnosed as undifferentiated cancer based on a forceps biopsy specimen. We attempted to minimize the selection bias by including consecutive patients identified from our database on ESD. 

## 5. Conclusions

In conclusion, the long-term outcomes of ESD for EGC-HH were favorable and comparable to those of PuD-EGC if the tumor met the curative endoscopic resection criteria. Therefore, ESD can be considered as an acceptable treatment option for EGC-HH meeting curative endoscopic resection criteria. The current Japanese criteria of non-curative ESD for EGC-HH need to be validated, as supporting evidence for these criteria is still sparse. 

## Figures and Tables

**Table 1:** Comparison of clinicopathologic characteristics of differentiated-type early gastric cancers with and without histological heterogeneity.

Variables	EGC-HH (n = 257)	PuD-EGC (n = 2386)	*p*-Value
Age (years)			0.159
Mean ± SD	61.9 ± 10.6	63.1 ± 9.8	
Median (range)	63 (27–86)	64 (31–90)	
Sex (%)			0.172
Male	192 (74.7)	1871 (78.4)	
Female	65 (25.3)	515 (21.6)	
Tumor site (%)			0.107
Antrum/angle	176 (68.5)	1778 (74.5)	
Body	75 (29.2)	558 (23.4)	
Fundus/cardia	6 (10.7)	50 (2.1)	
Tumor shape (%)			0.288
Elevated	153 (59.5)	1338 (56.1)	
Flat or depressed	104 (40.5)	1048 (43.9)	
Tumor size on pathology (cm)			<0.001
Mean ± SD	2.3 ± 1.1	1.5 ± 1.0	
Median (range)	2.0 (0.3–6.4)	1.3 (0.1–11.0)	
Tumor depth (%)			<0.001
Lamina propria	39 (15.2)	979 (41.0)	
Muscularis mucosae	108 (42.0)	1045 (43.8)	
SM1	38 (14.8)	176 (7.4)	
SM2 or SM3	72 (28.0)	186 (7.8)	
Differentiation (%)			<0.001
Well-differentiated	15 (5.8)	967 (40.5)	
Moderately-differentiated	230 (89.5)	1361 (57.0)	
Papillary adenocarcinoma	12 (4.7)	58 (2.4)	
Lymphatic invasion (%)			<0.001
Absent	182 (70.8)	2259 (94.7)	
Present	75 (29.2)	127 (5.3)	
Vascular invasion (%)			<0.001
Absent	246 (95.7)	2369 (99.3)	
Present	11 (4.3)	17 (0.7)	
Lateral margin (%)			<0.001
Negative	231 (89.9)	2321 (97.3)	
Positive/undetermined	26 (10.1)	65 (2.7)	
Vertical margin (%)			0.002
Negative	242 (94.2)	2332 (97.7)	
Positive/undetermined	15 (5.8)	54 (2.3)	

EGC-HH, differentiated-type-predominant early gastric cancer with histological heterogeneity; PuD-EGC, pure differentiated-type early gastric cancer; SD, standard deviation; SM1, submucosal invasion depth <500 µm from muscularis mucosa layer; SM2 or SM3, submucosal invasion depth ≥500 µm from muscularis mucosa layer.

**Table 2:** Comparison of short-term outcomes of endoscopic submucosal dissection of differentiated-type early gastric cancers with and without histological heterogeneity.

Variables	EGC-HH (n = 257)	PuD-EGC (n = 2386)	*p*-Value
En bloc resection (%)			0.163
Yes	248 (96.5)	2335 (97.9)	
No	9 (3.5)	51 (2.1)	
R0 resection (%)			<0.001
Yes	220 (85.6)	2286 (95.8)	
No or undetermined	37 (14.4)	87 (4.2)	
En bloc and R0 resection (%)			<0.001
Yes	216 (84.0)	2259 (94.7)	
No or undetermined	41 (16.0)	127 (5.3)	
Curative resection (%)			<0.001
Yes	126 (49.0)	2057 (86.2)	
No	131 (51.0)	329 (13.8)	
Perforation (%)			0.183
Absent	249 (96.9)	2341 (98.1)	
Present	8 (3.1)	45 (1.9)	
Bleeding (%)			0.651
Absent	244 (94.9)	2280 (95.6)	
Present	13 (5.1)	106 (4.4)	

EGC-HH, differentiated-type-predominant early gastric cancer with histological heterogeneity; PuD-EGC, pure differentiated-type early gastric cancer.

**Table 3:** Cases of differentiated-type-predominant early gastric cancer with histological heterogeneity with areas of undifferentiated component that exceed 20 mm in length or with undifferentiated component present in the submucosal invasion area.

Case	Age	Sex	Tumor Site	Shape	Pathologic Size (mm)	Depth	Pathology	UD Component	UD Component Length (mm)	UD Component in Submucosa	Recurrence	Recurrence-Free Survival (Months)
Case 1	53	M	Body	Elevated	46	MM	MD	PD, 30%	24	No	No	63
Case 2	45	F	Antrum	Depressed	40	MM	MD	SRC, 20%	24	No	No	84
Case 3	40	F	Antrum	Flat	32	LP	MD	SRC, 45%	24	No	No	63
Case 4	56	M	Body	Depressed	30	SM1	MD	PD, 20%	8	Yes ^a^	Op, LNM (-)	NA

M, male; F, female; MM, muscularis mucosae; LP, lamina propria; SM1, submucosal invasion depth <500 µm from the muscularis mucosa layer; MD, moderately-differentiated; UD, undifferentiated; PD, poorly differentiated; SRC, signet ring cell; Op, operation; LNM, lymph node metastases; NA, not applicable. ^a^ The length of the undifferentiated component in the submucosal invasion area was 1 mm.
